# Immune Regulation of Adult Neurogenic Niches in Health and Disease

**DOI:** 10.3389/fncel.2020.571071

**Published:** 2021-01-20

**Authors:** Sana Chintamen, Fatima Imessadouene, Steven G. Kernie

**Affiliations:** ^1^Neurobiology and Behavior, Columbia University Irving Medical Center, New York, NY, United States; ^2^Department of Pediatrics, Columbia University Irving Fefere Medical Center, New York, NY, United States

**Keywords:** neurogenesis, microglia, development, inflammation, neurodegeneration, cytokine, neuroimmune, pathology

## Abstract

Microglia regulate neuronal development during embryogenesis, postnatal development, and in specialized microenvironments of the adult brain. Recent evidence demonstrates that in adulthood, microglia secrete factors which modulate adult hippocampal neurogenesis by inhibiting cell proliferation and survival both *in vitro* and *in vivo*, maintaining a balance between cell division and cell death in neurogenic niches. These resident immune cells also shape the nervous system by actively pruning synapses during critical periods of learning and engulfing excess neurons. In neurodegenerative diseases, aberrant microglial activity can impede the proper formation and prevent the development of appropriate functional properties of adult born granule cells. Ablating microglia has been presented as a promising therapeutic approach to alleviate the brain of maladaptive immune response. Here, we review key mechanisms through which the immune system actively shapes neurogenic niches throughout the lifespan of the mammalian brain in both health and disease. We discuss how interactions between immune cells and developing neurons may be leveraged for pharmacological intervention and as a means to preserve adult neurogenesis.

## Introduction

Neurodegenerative diseases such as Alzheimer's and Parkinson's disease impair both longevity and quality of life. Hallmarks of these diseases are distinct pathology that cause irreversible damage or death to neurons and result in progressive cognitive and often physical decline. The progressive nature of neurodegenerative diseases is attributed largely to immune system dysregulation and chronic inflammation resulting in neuronal death (Glass et al., 2010; Ransohoff, 2016a; Chitnis and Weiner, 2017). Currently, there are no effective treatments to prevent and recover from the pathology, neuronal death, and cognitive decline seen in essentially all forms of neurodegenerative disease.

Exercise-induced neurogenesis enhances neurotrophic factor release which is felt to underlie the beneficial effects on cognition seen in animal models of age-associated neurodegenerative diseases and acquired brain injuries (Adlard et al., 2005; Erickson et al., 2010; Maass et al., 2016). While enhancement of neurogenesis via growth factors is promising, the role of immunomodulation in neurodegenerative diseases also presents challenges to neuronal maintenance and repair.

Immune cells are an integral part of the two main neurogenic niches in the adult brain—the subgranular zone of the hippocampus (SGZ) and the subventricular zone (SVZ) lining the lateral ventricles (Sierra et al., 2010; Ribeiro Xavier et al., 2015). During adulthood, homeostatic microglia comprise the majority of the immune population of this niche. These resident macrophages respond rapidly to both cues in the external physical environment, as well as changes in the molecular environment of the central nervous system (CNS). In neurodegenerative diseases, immune cells play an even more active role. They are the most motile cells in the brain and as its resident phagocytes, their role in clearing the brain of neurotoxic elements is essential for preventing neurotoxic elements from damaging neurons (Wolf et al., 2017; Li and Barres, 2018). Immune signaling also affects neurogenesis as neural stem and progenitor cells express receptors for many cytokines and immune molecules, suggesting they are susceptible to immune regulation in the adult brain (Ekdahl et al., 2009).

Microglia-neuronal crosstalk is a dynamic process that is reflected in the neurogenic niche throughout mammalian life (Butovsky and Weiner, 2018). The immune system in the brain, much like the immune system in the periphery, is a complex and dynamic element. This complexity affords the brain a specialized ability to respond to its diverse and evolving needs from development to the aged brain, adapting to change and challenges along the way (Ransohoff and Perry, 2009; Tchessalova et al., 2018). Dysfunction in both innate and adaptive cells have been linked to a host of neurodegenerative diseases in the central nervous system (Ransohoff, 2016a; Gan et al., 2018; Scheiblich et al., 2020). Thus, the maintenance of proper immune function is a great challenge in translational neuroscience and needs to be understood with great precision. Here, we describe the trajectory and development of neural stem cells in the adult brain from the perspective of immune regulation. We will focus on the effects of interactions between microglia and developing neurons in both health and disease, highlighting some key molecules that are common in various neurodegenerative disorders with known functions in regulating adult neurogenesis.

## Immune Regulation of Embryonic and Postnatal Neuronal Development

Derived from yolk sac progenitors in the mesoderm, microglia are the only glial cell type that does not originate from the rest of the neuroectoderm (Ginhoux et al., 2010). After migrating, they integrate with the CNS very early in development and persist throughout life. This highlights their importance in nervous system development (Reemst et al., 2016). Deficiency of microglial development and function is deleterious for CNS function and can lead to embryonic lethality or long-lasting cognitive deficits (Tong and Vidyadaran, 2016).

Microglia tightly regulate neurogenesis by promoting neuronal differentiation in dorsal forebrain progenitors (Nandi et al., 2012). Growth factors released by microglia are important for brain patterning. Colony Stimulating Factor 1 (CSF1) is important for tissue macrophages, and necessary for microglial proliferation and survival (Elmore et al., 2014). Lack of CSF1R signaling in progenitors not only results in increased proliferation, but also increased apoptosis in these cells. This provides evidence that CSF1R signaling via its ligands CSF1 and Il-34 are seen to be pro-neurogenic in development (Nandi et al., 2012). Immune activity is essential in promoting development of young neurogenic niches in early postnatal development, as microglia secrete cytokines required for the formation of neurons, and oligodendrocytes in the SVZ in a differential manner (Shigemoto-Mogami et al., 2014). As mammals generate more neurons than are needed, microglia presumably eliminate superfluous progenitors. They induce cell death in developing neural progenitors in the young brain in a Dap12/Cd11b-dependent manner as deficiencies in either Dap12 or Cd11b reduces superoxide production and apoptotic cells in the neonatal brain (Wakselman et al., 2008).

Finally, innate immune pathways such as the complement cascade, shape the brain during postnatal development. Microglia function to prune exuberant synapses during critical learning periods in a judicious manner, tagging synapses for elimination based on activity (Schafer et al., 2012). While phagocytic activity is high during development, as marked by immune activation and the presence of complement cascade proteins, they recognize and avoid synapses that should not be pruned (Lehrman et al., 2018). When microglia fail to prune superfluous synapses, development of normal cognitive and social behaviors are impeded. An experimental model of maternal immune activation illustrates the importance of interactions between immune dysfunction and developing neural circuits (Andoh et al., 2019). In this model, decreases in neurogenesis pair with inability of synapse elimination via phagocytosis to yield deficits in cognitive and social development. The deleterious effects of this can be rescued through exercise, which is known to enhance neurogenesis and increase beneficial microglial activity. When microglia are stimulated in this manner they clear excess synapses, allowing remaining synapses to strengthen and support learning. Thus, microglial activity instructs neurogenic development and conversely, developing neurons guide microglial activity.

## Immune Regulation of Adult Neurogenesis in Health

In adulthood, the brain becomes less plastic and relies on more stable connections. Microglial activity throughout the brain is shifted primarily toward surveillance, involving highly rapid process extension and retraction. In neurogenic niches, however, they continue to take part in shaping neurogenesis (Sierra et al., 2010; Ribeiro Xavier et al., 2015). Neurogenesis becomes limited to the SGZ and SVZ. Factors such as vascular endothelial growth factor (VEGF) secreted by neural progenitors can stimulate microglial activity, inducing proliferation and phagocytosis, supporting the view that reciprocal interactions guide neuronal development (Mosher et al., 2012). These are reflected in specialized properties that microglia maintain in neurogenic niches, including increased proliferation, amoeboid morphology, and greater lysosomal content (Marshall et al., 2014; Ribeiro Xavier et al., 2015; Kreisel et al., 2019).

### Immune Regulation of Activation and Proliferation in the Niche

Dormant stem cells arising from embryonic development reside in both neurogenic niches (Fuentealba et al., 2015; Berg et al., 2019). Cells from these pools are steadily activated and rapidly proliferate across adulthood.

After proliferation, the vast majority of progenitors do not survive to differentiate. These cells are rapidly cleared by microglia that are closely surveying the niche for apoptotic cells. This clearance occurs through engulfment by microglial processes followed by phagocytosis (Sierra et al., 2010). Whether immune cells actively trigger cell death in developing neurons or whether stem cells autonomously regulate their own fate in the SVZ and SGZ is subject to much debate. Microglia may actively elicit cell death in progenitors as they do in early postnatal development and in the cerebellum, or alternatively, they may simply clear the remains of cells which are committed to cell death themselves (Marin-Teva et al., 2004; Wakselman et al., 2008; Ayata et al., 2018).

Phagocytic activity is a core aspect of microglial physiology and function with different receptors and pathways that respond to different stiumuli (Zagorska et al., 2014; Fourgeaud et al., 2016). Unregulated phagocytosis may result in destruction or clearance of healthy neurons in disease (Galloway et al., 2019). While previously viewed as a passive mechanism to clear apoptotic neurons, it is now known that phagocytosis is part of a negative feedback loop which acts to regulate the neurogenic niche (Sierra et al., 2010; Diaz-Aparicio et al., 2020). Chronic inhibition of phagocytic pathways results in reduced neural progenitors in the adult brain. Temporary inhibition of phagocytosis, on the other hand, results in transiently increased proliferation of dividing progenitors. After engulfing apoptotic neurons, microglia undergo transcriptional reprogramming that results in a coordinated release of cytokines-including Il-6, TNF-α, and Il-1ß which are involved in pathways contributing to neurogenesis (Diaz-Aparicio et al., 2020).

Microglial input during development can have long lasting effects on the adult niche. Without CSF1R signaling, there is a higher rate of apoptosis in progenitors from both the SVZ and SGZ (Nandi et al., 2012). Even in adult niches, neural progenitor cells and immature neurons are sensitive to immune input. They express receptors for many cytokines and other immune molecules. Leukemia Inhibitory Factor which is transiently increased after injury promotes stem cell self-renewal (Bauer and Patterson, 2006). Il-6, which is increased in both systemic immune challenges and in neurodegenerative diseases, induces proliferation and self-renewal in neural stem cells (Storer et al., 2018). Progenitors also express C3aR and C5aR, receptors for complement cascade proteins. Perturbing C3aR signaling through either C3 ablation or receptor antagonist delivery results in reduced neurogenesis, which is worsened after ischemic injury (Rahpeymai et al., 2006). Thus, many cytokines transiently enhance the early stages of neurogenesis. Long-term perturbations in immune signaling results in decreased neurogenesis, potentially due to exhaustion of the progenitor population (Diaz-Aparicio et al., 2020). This may be a compensatory mechanism for neurons lost with inflammation. However, whether this increased proliferation results in productive neurons can vary depending on the duration and severity of the immune response.

Various stimuli including environmental enrichment and exercise stimulate activation and proliferation of neural stem and progenitor cells (Kempermann et al., 1997; van Praag et al., 1999). This increase is in part mediated by immune activity, which is required for an exercise-dependent increase in hippocampal neurogenesis (Ziv et al., 2006; Olah et al., 2009; Vukovic et al., 2012). Voluntary running leads to stimulation in microglial activity- marked by proliferation and differential gene expression (Ziv et al., 2006; Olah et al., 2009). Microglia increase proliferation via changes in fractalkine signaling (Vukovic et al., 2012). Thus, microglia are often mediators between peripheral stimuli and CNS response throughout mammalian life.

### Immune Regulation of Differentiation, Neuroblast Survival, and Neuronal Maturation

Microglia in neurogenic niches are specialized to support neuroblast differentiation, survival, and migration (Marshall et al., 2014; Ribeiro Xavier et al., 2015; Kreisel et al., 2019). In the hippocampus, failure of immature neurons to survive, migrate, and incorporate into synaptic circuits lead to deficits in hippocampal-dependent learning and memory (Deng et al., 2009; Vukovic et al., 2013). Therefore, survival and integration of immature neurons is a crucial step in maintaining certain forms of learning and memory.

Differentiation of progenitors to neuroblasts remains unaffected in the absence of microglial signaling following microglial depletion. Independent studies note no relative changes in the number of cells differentiating into neuroblasts or astrocytes, suggesting that homeostatic microglial input is not required for the early stages of neurogenesis (Kreisel et al., 2019; Kyle et al., 2019; Willis et al., 2020). Survival and migration, however, require interactions between immature neurons and microglia (Rahpeymai et al., 2006; Ribeiro Xavier et al., 2015; Kreisel et al., 2019).

Neurogenesis is often coupled with angiogenesis as developing neurons are in high need of growth factors and other support molecules for their development. One signaling molecule induced by this is VEGF, which stimulates endothelial proliferation and survival and leads to the formation of new blood vessels and enhances survival of doublecortin-expressing, immature neurons. Microglia in the dentate gyrus, unlike microglia elsewhere in the hippocampus, exclusively respond to VEGF stimulation and are required for mediating the VEGF-dependent increase in neurogenesis (Kreisel et al., 2019).

Healthy neurons communicate to microglia via release of fractalkine, also known as CX3CL1. Disruption of its receptor, CX3CR1 in microglia can also cause major deficits in hippocampal neurogenesis (Rogers et al., 2011). Decreased levels of CX3CR1 are associated with premature aging in microglia and result in elevated cytokines (Vukovic et al., 2012; Gyoneva et al., 2019). These same cytokines also appear in niches where neurogenesis is impaired.

In pathological states, induction of immune activation might perturb rates of differentiation to astrocytes vs. neurons (Kernie et al., 2001). In the dorsal SVZ, nestin-expressing neural progenitors express the receptor for Il-10, Il-10R1. Il-10 signaling specifically acts on this subset of progenitors and restricts their differentiation into neuroblasts (Perez-Asensio et al., 2013).

Immune input also regulates neuronal maturation and circuit properties of adult born neurons (Reshef et al., 2017; Wallace et al., 2020). Microglia have been known to participate in pruning of developing circuits, but evidence also shows they promote dendritic development (Paolicelli et al., 2011; Schafer et al., 2012; Parkhurst et al., 2013; Lehrman et al., 2018; Wallace et al., 2020). Disruption of microglial input to neurons in the process of maturing and incorporating into synapses can be deleterious to proper circuit function (Reshef et al., 2017; Wallace et al., 2020). Global depletion of microglia impacts neuronal maturation in the olfactory bulb by reducing response to odor stimuli, decreasing turnover of spines and altering electrophysiological properties of these cells when they fail to mature appropriately (Reshef et al., 2017; Wallace et al., 2020).

Without microglial elimination of weak synapses, these neurons have a greater density of filipodia spines, concurrent with smaller mushroom spines, further supporting the role of microglial input in dendritic spine development (Wallace et al., 2020). Weaker electrophysiological properties, in this case reduced amplitude of spontaneous excitatory post synaptic currents in adultborn granule cells impact the circuit at large, as mitral cell response is increased (Reshef et al., 2017; Wallace et al., 2020). Microglia-neuronal cross talk is essential for proper spine development, maturation, and synaptic incorporation of adult born granule cells in the olfactory bulb. Microglia input is necessary for every stage of neuronal development. Therefore, neuroinflammation and aberrant microglial activity that result in disruptions in microglial-neuronal crosstalk may explain cognitive symptoms associated with perturbed adult neurogenesis and neural plasticity in neurogenic niches.

## Immune Regulation of Adult Neurogenesis in Disease

Chronic neuroinflammation is linked to cognitive decline in most neurodegenerative diseases. Prolonged inflammation stems from aberrant activation of microglia and a failure of the immune system to resolve the inflammation. This is not only a significant challenge to developing neurons in neurogenic niches, but also an important target for therapeutic intervention (Biber et al., 2016). Neurogenic niches can transiently increase neurogenesis to compensate for loss of neurons, but this doesn't lead to the production of functional neurons, which can replace lost neurons (Monsonego and Weiner, 2003). To illustrate the relationship between neurodegeneration, inflammation, and neurogenesis we highlight two models of neurodegeneration, Alzheimer's Disease (AD) and traumatic brain injury (TBI) which have in common neuroinflammation and altered neurogenesis (Akiyama et al., 2000; Kernie et al., 2001; Acosta et al., 2013; Scopa et al., 2020). Additionally, TBI can predispose patients to AD, other neurodegenerative disorders, and neuropsychiatric disorders (Rogers and Read, 2007; Dams-O'Connor et al., 2016). These comorbidities are associated with sustained inflammation, providing further links between common pathologies seen in different neurodegenerative diseases (Newcombe et al., 2018).

### Microglial Activation Following Neurodegeneration

Aging is one of the most significant risk factors in age-associated neurodegenerative diseases (Hou et al., 2019). Age-associated decline of neurogenesis, can in part be attributed to aberrant immune activity (Dulken et al., 2019; Wu et al., 2020). With aging, the neurogenic niche undergoes changes in cellular composition. The largest population in the niche become cells expressing high levels of Cd45, or non-microglia immune cells (Artegiani et al., 2017). There is a decrease in neuroprotective cytokines and an increase in neurotoxic cytokines (Vukovic et al., 2012). Infiltration of T-cells in neurogenic niches leads to release of If-γ which results in a neurotoxic environment, contributing to reduced neurogenesis (Dulken et al., 2019). In the aged brain, decline of brain derived neurotrophic factor (BDNF) correlates to microglial activation and deleterious effects on neurogenesis (Wu et al., 2020). This precedes the development of Alzheimer's Disease whose pathology can be alleviated with exercise-induced neurogenesis through release of BDNF (Choi et al., 2018).

Neuronal death or damage creates a toxic environment for surrounding healthy neurons (Takahashi et al., 2005). As the resident phagocytes and first responders to cytotoxic elements, microglia adopt a damage-associated phenotype which has the potential to prevent further cell death (Krasemann et al., 2017). Damage-associated microglia upregulate genes associated with phagocytosis and lipid metabolism (Keren-Shaul et al., 2017; Krasemann et al., 2017). In aging and neurodegenerative diseases, microglia may lose their efficacy to remove toxic elements, and in some cases even adopt a neurodegenerative phenotype (Krasemann et al., 2017). Neurodegenerative microglia exhibit transcriptional and functional phenotypes associated with perturbed lipid metabolism in genes whose disruption are known risk factors in AD. Breakdown of these mechanisms results in lipid-droplet accumulation and in proinflammatory phenotypes in disease models and in the human brain (Ransohoff, 2016a; Marschallinger et al., 2020).

These maladaptive phenotypes comprise gene networks largely controlled by the ApoE-Trem2 pathway- genes with variants that are associated with a higher risk in developing late onset AD as well as unfavorable outcomes after TBI (Jordan, 2007; Castranio et al., 2017; Efthymiou and Goate, 2017; Krasemann et al., 2017; Tensaouti et al., 2020).

Dysregulation of microglial and immune activity is a consistent and prevalent theme in neurodegeneration. Microglia activated in this aberrant manner promote inflammation, creating an altered environment in which developing neurons are especially susceptible to cell death or reduced neurogenesis (Ekdahl et al., 2003, 2009; Monje et al., 2003; Yu et al., 2008). Chronic inflammation has also been linked to cognitive decline (Kohman and Rhodes, 2013).

### Adult Neurogenesis Following Neuroinflammation

Inflammation is a specialized immune response to a foreign substance or to damage. In the brain, this is characterized by the release of cytokines, chemokines, and secondary messenger proteins by microglia and astrocytes (Carson et al., 2006). Secreted immune-cell molecules are categorized as pro-inflammatory or anti-inflammatory cytokines (Jun-Ming and Jianxiong, 2007). Cytokines that are associated with inflammation are upregulated in neurodegenerative diseases such as Alzheimer's Disease, Parkinson's Disease, ischemic stroke, and traumatic brain injury (Alzheimer's, 2016; Dugue and Barone, 2016; Guzman-Martinez et al., 2019). If the inflammation is left unresolved, there are deleterious consequences which compromise the survival and integrity of neural circuitry in the brain and prevent the successful incorporation of adult born neurons (Belarbi et al., 2012). As neuroinflammation is coincident with neurodegeneration, its link to neurogenesis is crucial to understanding how these diseases affect adult neurogenesis (Guzman-Martinez et al., 2019).

An example of a neurological disorder which consists of neuroinflammation, neurodegeneration, and perturbed neurogenesis is Alzheimer's Disease (AD), currently the most prevalent cause of dementia. It involves two major pathologies—amyloid beta plaque accumulation and neurofibrillary tangles—both of which contribute to neurodegeneration and cognitive decline (Alzheimer's, 2016). Most genetic risk factors identified are genes participating in immune function, sparking interest in targeting the immune system as a means of therapeutic intervention (Monsonego and Weiner, 2003; Karch and Goate, 2015; Biber et al., 2016). In fact, both forms of pathology in AD are accompanied by inflammation. Some key cytokines implicated in exacerbating the pathology include TNF-α,Il-1ß, Ifn-γ, and Il-6 (Kinney et al., 2018). Along with immune function dysregulation, adult neurogenesis is also impaired in human patients suffering from AD (Moreno-Jimenez et al., 2019).

Microglia adopting a pro-inflammatory phenotype were initially believed to prevent neurogenesis, and therefore anti-neurogenic. Upon systemic endotoxin injection, used to model an in immune challenge resulting in microglia activation, an inverse correlation between activated microglia and neurogenesis was observed. This decrease in neurogenesis was rescued with anti-inflammatory drugs (Ekdahl et al., 2003; Monje et al., 2003). Accumulating evidence demonstrates that these pro- and anti-inflammatory phenotypes do not directly translate into anti- or pro- neurogenic actions, for developing neurons (Ekdahl et al., 2009; Fuster-Matanzo et al., 2013; Ransohoff, 2016b).

For example, Il-10 has been classically considered an anti-inflammatory cytokine, yet it inhibits differentiation of neural progenitors into neurons and is therefore anti-neurogenic (Lobo-Silva et al., 2016). Conversely, interferon-γ (Ifn-γ) encourages both neural progenitor proliferation and neuronal differentiation especially in aged mice or mice with Alzheimer's Disease-like pathology. This suggests a neuroprotective and pro-neurogenic role in the context of neurogenesis resulting from aging or neurodegeneration despite its classification as a pro-inflammatory cytokine (Baron et al., 2008). Tnf-α displays pleiotropic effects depending on the receptor it binds to. TNFR1 signaling results in reduced proliferation in the adult dentate gyrus while TNFR2 promotes neurogenesis (Iosif et al., 2006; Chen and Palmer, 2013). This altered environment has significant consequences for the neurogenic niche and does not always align with traditional understanding of cytokine function (Ekdahl et al., 2009; Fuster-Matanzo et al., 2013; Ransohoff, 2016b).

### Therapeutic Implications

While anti-inflammatory drugs can attenuate deficits in murine adult neurogenesis, the mechanisms mediating their effects are not entirely understood and their application in humans less effective or even detrimental (Wyss-Coray and Mucke, 2000; Boehme et al., 2014; Scott et al., 2018). Instead, over the last few years, microglial ablation via pharmacological inhibitors of CSF1R have emerged as a potential method to rescue the deleterious effects of aberrant microglia activation and neuroinflammation. These inhibitors allow for the reversible ablation of microglia from the brain (Elmore et al., 2014; Rice et al., 2015). In a model of neuronal death, microglia exhibit elevated cytokines, a characteristic feature of immune activation and inflammation. Loss of neurons can be compensated by increased synaptogenesis in surviving neurons. However, for this to occur, microglial synaptic pruning must be inhibited via microglial depletion. This paradigm appears to restore cognitive function and behavior. Microglia depletion was suggested as a potential therapeutic strategy in other forms of neurodegeneration (Rice et al., 2015). This study raised an interesting proposition, that microglial ablation could rescue the deleterious effects of aberrant microglia activation and subsequent inflammation.

In traumatic brain injury (TBI), a model of injury-induced neurodegeneration, Willis and colleagues address the dynamics of microglial input on neural damage and subsequent repair. TBI itself results in deficits in learning and memory concomitantly with decreased doublecortin (DCX)-expressing cells (Willis et al., 2020). Microglial depletion alone in this model reduces survival of DCX-expressing, immature neurons. However, repopulation following depletion results in attenuation of neuronal loss compared to TBI mice without treatment. These cellular differences translate to functional differences in behavior as different behavioral tests show impaired learning and memory. These effects are time dependent as there is a limited temporal window after injury for which microglia turnover is beneficial. Specifically, microglia turnover needs to be coincident with the acute insult resulting from TBI (Willis et al., 2020).

The restorative effects of microglial replenishment create a permissive environment for survival of doublecortin cells which are implicated in forms of learning and memory attributed to spatial navigation and working memory (Blaiss et al., 2011; Willis et al., 2020). Furthermore, inducing local depletion followed by repopulation also achieves better cognitive outcomes. The traditionally pro-inflammatory cytokine Il-6 mediates these effects. Il-6 exhibits pleiotropic effects on adult neurogenesis under various contexts and levels of this cytokine are often elevated during enhanced neurogenesis and during postnatal development (Storer et al., 2018). In the case of the neuroprotective effects of repopulating microglia, Il-6 trans signaling between microglia and developing neurons is critical (Willis et al., 2020). Similar to its effects in TBI, microglia turnover in the aged brain using CSF1R inhibition results in restorative effects on neuronal function and cognition. Turnover of microglia using depletion and repopulation, in effect, revitalizes the immune compartment of the CNS by promoting neurogenesis and synaptogenesis (Elmore et al., 2018).

Depletion studies have elucidated key aspects of the immune response to brain injury. First, cognitive deficits are not necessarily caused by aberrant microglia activity but rather loss-of-function of microglia that would normally support survival of neuroblasts with injury or other inflammatory conditions (Elmore et al., 2018; Willis et al., 2020). This suggests there is a saturation of microglial clearance that can only be relieved with turnover of microglia (Willis et al., 2020). Secondly, this model supports the pleiotropic effects of cytokines traditionally associated with inflammation. Lastly, microglia turnover localized specifically to the neurogenic niche is sufficient to bolster survival of developing neurons upon injury (Willis et al., 2020).

Under homeostatic conditions, the neurogenic pool is able to regulate and self-maintain despite microglial depletion (Kreisel et al., 2019; Kyle et al., 2019; Willis et al., 2020). These mechanisms, however, are not selective and affect other cell types. For instance, CSF1R antagonism affects astrocytes. Drug treatment results in astrogliosis as marked by increased GFAP and S100 mRNA as well as protein levels without increases in cell number (Elmore et al., 2014, 2018). Additionally, microglial depletion affects functional properties developing granule cells originating in the SVZ (Reshef et al., 2017; Wallace et al., 2020). Therefore, pharmacological replacement of microglia as a form of treatment will require more specificity in focused areas to avoid off-target effects.

## Conclusion

Microglia dysfunction has been implicated in most neurological diseases which affect adult neurogenesis. Proper function of microglial signaling is crucial during development for healthy nervous system formation. This signaling is important for the patterning of the stem and progenitor pools that eventually shape the neurogenic niches in adulthood. Disruptions of appropriate microglial signaling due to genetic defects during development can result in long lasting effects on adult neurogenesis. Perturbations in microglial input to cells that have committed to a neuronal fate greatly affects neuroblast survival, ultimately leading to decreased neurogenesis. Defects at this stage may explain cognitive deficits associated with immune activation in many neurodegenerative diseases. Immune activity is therefore a potential target to modulate pathological outcomes and ameliorate the effects of inflammation on adult neurogenesis. Microglial turnover appears promising as a method of using endogenous repair mechanisms to bolster adult neurogenesis. As many immune molecules have opposing effects on different stages of neurogenesis and different areas of the brain, the timing and localization of microglial turnover to key timepoints in neurogenic niches may act to preserve and potentially enhance adult neurogenesis in health and disease.

## Author Contributions

SC and FI drafted manuscript. SC generated figure. SC and SK edited manuscript. All authors contributed to the article and approved the submitted version.

## Conflict of Interest

The authors declare that the research was conducted in the absence of any commercial or financial relationships that could be construed as a potential conflict of interest.
